# Guided Treatment of *Helicobacter pylori* Infections with Non-Invasive PCR Tests—The Glory Days of Primary Care?

**DOI:** 10.3390/jcm11154320

**Published:** 2022-07-25

**Authors:** Maxime Pichon, Bernard Freche, Christophe Burucoa

**Affiliations:** 1 CHU Poitiers, Infectious Agents Department, Bacteriology Laboratory, 86021 Poitiers, France; christophe.burucoa@chu-poitiers.fr; 2 INSERM U1070 Pharmacology of Antimicrobial Agents and Antibiotic Resistance, University of Poitiers, 86073 Poitiers, France; bernard.freche@univ-poitiers.fr; 3 University of Poitiers, Department of General Medicine, Faculty of Medicine and Pharmacy, 86073 Poitiers, France

*Helicobacter pylori*, a gram-negative bacterium that specifically colonizes gastric mucosa, is one of the most widespread infectious diseases in the world. More than half of the world population (estimated at more than four billion in 2015) are infected and an estimated 30 million people worldwide receive eradication therapy to treat *Helicobacter pylori* [1]. The vast majority receive empirical treatment: either triple proton pump inhibitor therapy plus amoxicillin–clarithromycin (or quadruple therapy when combined with metronidazole), or quadruple proton pump inhibitor therapy plus metronidazole–tetracycline combined with bismuth salts [2]. Due to the high prevalence of resistance to clarithromycin (the key antibiotic in eradication treatments), which is responsible for the therapeutic failures of triple therapy, it is now recommended, in the many countries where the prevalence of primary resistance to clarithromycin exceeds 15%, to abandon empirical treatments in favor of guided therapy [3,4]. The latter is guided by phenotypic antibiotic susceptibility testing or genotypic molecular biology (PCR targeting clarithromycin resistance mutations) [2,5,6,7]. Guided therapy is widely recognized as a more effective and efficient strategy than empirical therapy insofar as it prevents the spread of resistance and preserves patients’ digestive microbiota [8,9].

While the cure for *H. pylori* after treatment can be assessed using the 13C-urea breath test (UBT) or stool antigen test at least 4 weeks after treatment, until recently, guided therapy necessitated endoscopic diagnosis with biopsy-based methods that required the intervention of gastroenterologists [10]. The recent development and excellent performance of several real-time PCR techniques to detect *Helicobacter pylori* infection and clarithromycin resistance mutations in non-invasive samples (patient stool) provide general practitioners with a tool that can be used in routine diagnostic situations [11]. Indeed, as these tests now enable the efficient screening and diagnosis of *Helicobacter pylori* infections in their patients, they remove a barrier to the implementation of a guided treatment strategy and eliminate the need for routine referral to a gastroenterologist. The non-invasive nature of these tests is conducive to self-sampling at home. Moreover, transport media allow the preservation of nucleic acids at room temperature during the several days necessary for the postal transport of the sample, before analysis in a specialized laboratory. This approach has demonstrated its effectiveness in getting patients to adhere to the procedure [12].

Already used to analyze patients with indications for *Helicobacter pylori* screening and eradication, this test will limit the referral of non-*Helicobacter pylori* infected patients to a gastroenterologist [2,7,13]. Only patients older than 45 years and/or with a specific request for pre-cancerous histological lesions (e.g., personal history of symptoms suggestive of Helicobacter pylori, and/or family history of neoplasia) should be referred for endoscopy. Reduction in inappropriate referrals will help achieve the goal of five biopsies performed, as recommended.

After the implementation of this innovative test, primary care physicians will be able to manage their patients more comprehensively and effectively by prescribing the investigation and eradication of Helicobacter pylori infection. In such a procedure, UBT remains of major interest to assess the efficacy of eradication therapy (serology remains positive long after infection and cure, and PCR may be falsely positive, due to persistent DNA and non-infectious strains) [14]. Furthermore, to optimize the cost-effectiveness of the procedure, and given the relatively high cost of molecular biology, a two-step approach could be imagined, based on initial screening by serology, after which only positive patients would be tested by molecular biology in the stool. In this Special Issue of the *Journal of Clinical Medicine*, entitled “*Helicobacter pylori* Infection and Other Bacterial Infectious Diseases: Complications, Diagnosis and Therapeutic Management”, we described the protocol of an ongoing French multicenter study, which aims to evaluate the performance of different approaches to improve the efficacy of *Helicobacter pylori* eradication therapy [15]. We plan to compare usual patient management, including referral to a gastroenterologist, versus an innovative approach in which general family practitioners lead the whole process, from the diagnosis through to the introduction of a guided treatment to eradication control. Secondary objectives will consist of confirmation, in the context of routine disease management by the family practitioner, of the different biological tests (i.e., serology, stool PCR and UBT) [12].

Finally, it is important to remember that, compared to targeted biopsies, stool diagnosis, whether or not by molecular biology, is not only non-invasive, but also has a major benefit with regard to antibiotic resistance. Indeed, even if the infecting populations are still present in all the antrum and fundus samples of infected patients (as described by Pichon et al. [16]), the minority subpopulations are not uniformly distributed in this mucosa (some develop preferentially in the antral part of the mucosa, while others are in the fundic part), which underlines a risk of false negative research of resistant populations (manuscript in preparation).

In conclusion, thanks to the performances achieved, in the context of pauci-symptomatic and pre-neoplastic disease caused by *Helicobacter pylori*, primary care physicians are in the front line to establish the indications for the search and eradication of *Helicobacter pylori*. In France, it has been estimated that they see the entire population at least once a year. This approach would benefit from strong adherence in the general population and could receive as strong an adherence as cryptic hemorrhages caused by colorectal neoplasia. The golden age of general medicine should therefore make us rethink the global management of patients infected or suspected to be infected by *Helicobacter pylori*, the objective being to significantly decrease the dramatic number of deaths caused by gastric cancer in France, and, after standardization to adapt to other countries according to their specificities, eventually, in countries worldwide, following the issuance of global recommendations. Has the time come to publish new Maastricht recommendations that include the general practitioner as the management leader?

## References

[1] Bravo D., Hoare A., Soto C., Valenzuela M.A., Quest A.F. (2018). *Helicobacter pylori* in Human Health and Disease: Mechanisms for Local Gastric and Systemic Effects. World J. Gastroenterol..

[2] Malfertheiner P., Megraud F., O’Morain C.A., Gisbert J.P., Kuipers E.J., Axon A.T., Bazzoli F., Gasbarrini A., Atherton J., Graham D.Y. (2017). Management of *Helicobacter pylori* Infection-the Maastricht V/Florence Consensus Report. Gut.

[3] Bouilhat N., Burucoa C., Benkirane A., El Idrissi-Lamghari A., Al Bouzidi A., El Feydi A., Elouennas M., Benouda A. (2015). High-Level Primary Clarithromycin Resistance of *Helicobacter pylori* in Morocco: A Prospective Multicenter Molecular Study. Helicobacter.

[4] Raymond J., Lamarque D., Kalach N., Chaussade S., Burucoa C. (2010). High Level of Antimicrobial Resistance in French *Helicobacter pylori* Isolates. Helicobacter.

[5] Megraud F., Bruyndonckx R., Coenen S., Wittkop L., Huang T.-D., Hoebeke M., Bénéjat L., Lehours P., Goossens H., Glupczynski Y. (2021). *Helicobacter pylori* Resistance to Antibiotics in Europe in 2018 and Its Relationship to Antibiotic Consumption in the Community. Gut.

[6] Burucoa C., Axon A. (2017). Epidemiology of *Helicobacter pylori* Infection. Helicobacter.

[7] De Korwin J.-D. (2013). New recommendations for the diagnosis and the treatment of *Helicobacter pylori* infection. Presse Med..

[8] Delchier J.-C., Bastuji-Garin S., Raymond J., Megraud F., Amiot A., Cambau E., Burucoa C., HELICOSTIC Study Group (2019). Efficacy of a Tailored PCR-Guided Triple Therapy in the Treatment of *Helicobacter pylori* Infection. Med. Mal. Infect..

[9] Wenzhen Y., Yumin L., Quanlin G., Kehu Y., Lei J., Donghai W., Lijuan Y. (2010). Is Antimicrobial Susceptibility Testing Necessary before First-Line Treatment for *Helicobacter pylori* Infection? Meta-Analysis of Randomized Controlled Trials. Intern. Med..

[10] Kim S.E., Hwang J.H. (2022). Management of *Helicobacter pylori* Infection: A Comparison between Korea and the United States. Gut Liver.

[11] Marrero Rolon R., Cunningham S.A., Mandrekar J.N., Polo E.T., Patel R. (2021). Clinical Evaluation of a Real-Time PCR Assay for Simultaneous Detection of *Helicobacter pylori* and Genotypic Markers of Clarithromycin Resistance Directly from Stool. J. Clin. Microbiol..

[12] Pichon M., Pichard B., Barrioz T., Plouzeau C., Croquet V., Fotsing G., Chéron A., Vuillemin É., Wangermez M., Haineaux P.-A. (2020). Diagnostic Accuracy of a Noninvasive Test for Detection of *Helicobacter pylori* and Resistance to Clarithromycin in Stool by the Amplidiag *H. pylori* + ClariR Real-Time PCR Assay. J. Clin. Microbiol..

[13] Lamarque D., Burucoa C., Courillon Mallet A. (2017). Recommandations Sur Le Traitement de l’infection à *Helicobacter pylori* Chez l’adulte. Hépato-Gastro Oncol. Dig..

[14] Losurdo G., Francioso F., Pricci M., Girardi B., Russo F., Riezzo G., D’Attoma B., Bleve M.A., Iannone A., Celiberto F. (2022). A Prospective Study on *Helicobacter pylori* Rapid Urease Test False Negativity: Is It Time for Its Use in Restricted Situations?. Minerva Gastroenterol..

[15] Pichon M., Freche B., Burucoa C. (2022). New Strategy for the Detection and Treatment of *Helicobacter pylori* Infections in Primary Care Guided by a Non-Invasive PCR in Stool: Protocol of the French HepyPrim Study. JCM.

[16] Pichon M., Tran C.T., Motillon G., Debiais C., Gautier S., Aballea M., Cremniter J., Vasseur P., Tougeron D., Garcia M. (2020). Where to Biopsy to Detect *Helicobacter pylori* and How Many Biopsies Are Needed to Detect Antibiotic Resistance in a Human Stomach. J. Clin. Med..

